# Supplementary material for: Are carbon emissions trading and green financial instruments synergistic? -Comprehensive quantitative research based on content analysis
Source: PLoS One. 2024 Mar 7;19(3):e0298601. doi: 10.1371/journal.pone.0298601 (PMC10919601; doi:10.1371/journal.pone.0298601)
Supplement: S1 Data — (ZIP) [file pone.0298601.s001.zip › Data/地级市Z_F工作报告.zip!/░▓╗╒/╗┤▒▒╩╨/└┤╫╘╣1⁄2╓┌║┼í╛┬φ┐╦╩2╛▌═°í┐_2006.docx]

各位代表：

　　我代表市人民政府向大会作政府工作报告，请予审议，并请各位政协委员和其他列席人员提出意见。

　　一、二OO五年及“十五”时期的工作回顾

　　刚刚过去的一年，市政府在市委的领导和市人大、市政协的监督支持下，团结和带领全市人民，坚持以邓小平理论和“三个代表”重要思想为指导，认真贯彻党的十六大和十六届五中全会精神，全面落实科学发展观，紧紧围绕“争先进位、率先崛起”的要求，抢抓机遇、加快发展，较好地完成了市十三届人大三次会议确定的各项目标任务。

　　（一）经济持续较快增长，运行质量进一步提高。预计，2005年全市实现生产总值200亿元，增长12%；财政总收入完成26.5亿元，增长29%；城镇居民人均可支配收入8600元，增长18％；农民人均纯收入2650元，与上年基本持平；城乡居民储蓄存款余额141亿元，增长16.3％。工业经济支柱地位更加稳固。全市规模以上工业企业完成增加值99亿元，增长13%；实现利税28亿元，增长18%；综合效益指数达120%，呈现出总量攀升、速度加快、效益提高的运行态势。县区工业活力增强，不同所有制企业全面发展；股份制企业、“三资”企业生产继续向好，增加值分别增长13.2%和36.4%，工业经济成为推动全市加快发展的主要动力。农业综合生产能力稳步提升。面对秋季作物严重受灾、养殖业受禽流感疫情影响等不利因素，通过优化种植结构、发展规模养殖、扩大劳务输出、延长农业加工链条、活化市场流通等措施，农业和农村经济保持了稳步健康发展的良好态势，实现增加值22亿元。优质粮、优质棉、优质果蔬、优质经果林、畜牧水产五大主导产业进一步发展壮大，建成了100万亩优质麦、30万亩果蔬、100万头优质肉猪、塌陷区养殖和石榴标准化生产等一批具有区域竞争力的优势农产品基地，其中肉、蛋产量分别增长8%和6.4%。劳务经济不断发展，新增劳务输出5万人，新增劳务收入3.2亿元。农业产业化进程加快，具有较强经济实力和辐射带动能力的农业产业化龙头企业达120余家，专业批发市场25个；农村专业合作经济组织140多个，位居全省先进行列；加工业和商贸流通业健康发展，粮食及饲料加工业走在全省的前列。农业生产条件不断改善，有效灌溉面积达122万亩，综合机械化水平达85%。第三产业发展速度加快。预计全年实现增加值61亿元，增长13%。突出发展商贸流通，积极引进超市等各类连锁企业，并向农村延伸；住房等商品消费较快增长，旅游业发展势头较好，南湖湿地公园被批准为国家城市湿地公园，矿山公园已经通过国家评审委员会审查，相山风景区、龙脊山风景区正在申报AAA旅游区。

　　（二）投资规模继续扩大，发展后劲进一步增强。顺应国家产业政策导向，以实施省“86l”行动计划为重点，完善推进机制，加大投资力度，预计完成固定资产投资84亿元，增长31%。一批对全市经济社会发展具有牵动作用的大项目取得重大进展，大唐淮北电厂8#机组竣工投产；许疃等矿井扩建，涡北等矿井续建，飞亚高档服装面料等在建项目进展顺利，部分已竣工投产；总投资20亿元的煤焦化电项目一期工程、临涣选煤厂扩建项目开工建设；总投资95亿元的平山电厂项目和总投资7.2亿元的500千伏变电工程即将开工；国安电力二期前期工作扎实推进；南坪煤化工一期50万吨甲醇项目正在进行开工准备；总投资4.2亿元的采煤沉陷区综合治理项目开始实施；“淮水北调”项目列入省“十一五”重大项目建设规划。

　　（三）城市规划建设管理全面加强，创建成效进一步显现。城乡规划体系逐步完善实施。《淮北市城市总体规划（2003—2020）》基本完成，已报国务院审批；按照建设“双百双宜”城市的目标，《淮北市城市近期建设规划（2006—2010）》全面展开，完成了绿地系统、城市防洪等专业规划和人民路两侧、城市南部中心区、温哥华城新区、采煤沉陷区综合治理搬迁居住区等控制性详规；继续实施主城区“东进、西延、南扩”，城市建成区达65平方公里。基础设施建设得到加强。积极筹措建设资金，获得国家开行贷款7.45亿元；大力推进国有土地资本运营，筹措资金3.8亿元。建设城市基础设施项目21项，完成投资1.8亿元；改造了濉溪路，完成了人民东路、环山西路、翠峰南路、相山南路、淮海西路、惠民路、董庄路绿化和东外环路亮化等16项工程；开工建设了南湖路、南黎东路、3万人体育场、城市垃圾发电项目等工程；区域供水工程以及污水管网工程完成管道铺设62公里，基本建成高岳水厂、相南水厂；城市集中供热工程进一步推进，新建小区已实现集中供热。商贸服务建设项目逐步完善，商业步行街开街，盛世商贸城二期、东湖五金商贸城等主体工程完工。加快旧城改造和新区建设，完成投资8.5亿元，建设重点房地产项目17个，总面积160万平方米，其中启动旧城改造项目12个，动工面积66.5万平方米，经济适用房项目南黎花园等已开工建设，市民居住条件进一步改善。城市管理进一步规范。成立了城市管理行政执法局，相对集中行使行政处罚权，完成了市、区环卫一体化管理体制改革，城市管理水平明显提高。不断加大社区建设力度，选派优秀年轻干部到社区挂职，招聘了一批年轻的社区专干，改建、插建、购进社区居委会用房20处、6700多平方米，较好地解决了“有人办事、有房办事、有钱办事、有章理事”问题。创建工作成效显著。集中开展“五项治理”、“八项督查”和创建“文明示范街”等活动，我市再次被评为“全国创建文明城市工作先进城市”；创建全国园林城市工作成效显著，创建全国绿化模范城市活动深入开展，建城区人均公共绿地面积达到11.8平方米，森林覆盖率为14.9%。

　　（四）改革开放步伐不断加快，发展活力进一步激发。国企改革大力推进。以产权制度改革为主线，积极推进国有企业改革，市属国有企业改制面达65％；通过拍卖等多种形式变现资产，多方筹集资金1.6亿元，安置职工1.3万人；积极推进优势企业建立现代企业制度，飞亚纺织成功上市，募集资金1.36亿元。体制改革继续深化。深化政府机构改革，完成市发展和改革委员会、经济委员会（国资委）和中小企业发展局、商务局等部门的整合与组建工作。加强土地、技术、劳动力等要素市场建设，规范有形建筑、土地交易和产权交易市场。以建立公共财政体制为目标，不断调整优化支出结构，科技、教育、农业、社保等公共支出逐年增加。深化农村税费改革，认真落实各项惠农政策，全市免征农业税5075万元，发放粮食直接补贴、小麦良种补贴等各类涉农补贴4526万元。对外开放不断扩大。努力克服人民币升值、贸易壁垒等多种因素的影响，完成外贸进出口总额5300万美元，其中出口4500万美元。大力实施东向战略，继续加大招商引资工作力度，突出面向江浙和对韩招商，设立了驻温州办事处，与韩国抱川市的合作交流进一步加强，全年新批外商投资企业9家，实际利用外资2650万美元；引进省外资金28.1亿元，增长39.4%。出台《关于进一步加快淮北经济技术开发区建设和发展的决定》，推动市经济开发区和县区工业园区建设。非公有制经济不断发展。出台了《关于促进全市非公有制经济发展的若干意见》，初步建立了有利于非公有制经济发展的政策环境；开展银企对接活动，各商业银行与中小企业签订贷款项目82个，资金总额6.2亿元，已发放贷款3.2亿元，有力地促进了非公有制经济发展，增加值达50亿元。

　　（五）切实维护群众利益，和谐社会建设进一步加快。在全省率先出台了《关于我市加快构建和谐社会若干问题的意见》，突出抓好就业和再就业、社会保障、公共服务、安全防控等关系人民群众尤其是困难群众切身利益的问题。继续加大社会保障投入。全年社会保障方面的支出达4.7亿元，占财政总支出的32%。其中，新增城市医疗救助、资助困难家庭子女入学、儿童计划免疫、妇女儿童救助等方面的资金1032万元；全年发放低保金5154.7万元，实现了动态管理下的应保尽保；城市低保标准由191元提高到210元，低保水平居全省前列；制定出台了《淮北市建立和完善农村特困群众生活救助制度实施方案》，努力保障农村特困群众的基本生活，累计拨付特困救助金和救灾款762万元。努力扩大就业和再就业。选聘临时就业困难的100名应届大学毕业生到企业、乡村和社区工作；政府购买公益性岗位，安置下岗职工500名；大力开发就业岗位，新增城镇就业岗位2.7万个，9000余名下岗失业人员实现了再就业。高度重视社会稳定。建立健全全社会预警体系和应急机制，提高了保障公共安全和处置突发事件的能力。全面落实安全生产责任制和责任追究制，以煤矿、非煤矿山等为重点，排查和整治了一批安全事故隐患，努力防止重特大安全生产事故发生；强化社会治安综合治理，组织开展命案侦破、农村治安整治等专项行动，破获了一批有影响的大要案，保障了人民生命财产安全；“四五”普法和基层司法所规范化建设取得明显成效；加强群众来信来访和人民调解工作，组织开展了领导干部开门接访、带案下访活动，认真解决农村征地、城镇拆迁、企业改制等影响群众利益的突出问题，信访工作连续6年进入全省前3名。　

　　(六)统筹经济社会发展,社会事业进一步繁荣。科教兴市战略继续扎实推进。鼓励科技创新,重奖有突出贡献的科技人员和科研成果;多渠道筹集资金4000万元支持民营科技园建设;全市高新技术产业技工贸总收入15.2亿元;农业科技贡献率显著提高,彩色棉和专用棉培育、胚胎移植生物工程应用、标准化生猪饲养等10多个领域居全省领先水平。继续把教育放在优先发展的位置,不断巩固义务教育,加强素质教育,教育质量进一步提高;淮北一中新校区动工兴建,市一小温哥华城分校正式招生,淮北工业学校成为国家级重点中专学校,消除农村中小学危房面积近2.7万平方米。全面实施三项基本国策。扎实开展“两项清理”,夯实基层基础,人口和计生工作整体水平有所提高,较好完成了与省政府签订的目标。严格执行耕地保护和土地节约集约利用政策,努力保障全市经济社会发展的用地需求。加强环境保护工作,推行清洁生产,强化环境现场监察,整治企业违法排污行为,严厉查处“十五小”、“新五小”污染反弹。实施“引闸入化”工程,化家湖水库成功蓄水,有效改善了生态环境,解决了东部地区人畜饮水困难。各项社会事业全面进步。成功举办淮北市首届职工文化艺术节,群众性文化活动异彩纷呈,文化市场不断繁荣;图书馆新馆建设立项及选址等前期准备工作基本完成;广播电视事业健康发展,广电中心大楼开始筹建。卫生事业整体推进,市卫生大厦主楼全面封顶,市传染病医院改造完工,引入民资股份制改造了市妇幼保健院,在全省率先放开医疗市场准入,疾病控制工作有条不紊。全民健身运动蓬勃开展,竞技体育水平稳步提升,网球馆、田径训练场等项目竣工投入使用。继续整顿市场秩序,深入开展价格诚信活动,加大食品药品安全专项整治力度,查处药品案件187件。“双拥”工作扎实推进,国防教育、民兵应急队伍和人防工作进一步加强。解放军、武警官兵和广大民兵在救灾抢险等急难险重任务中发挥了重要作用。认真实施妇女儿童发展纲要,完成阶段性目标任务。统计工作质量和服务水平进一步提高,第一次全国经济普查工作圆满完成,并获得国家级先进称号。青年工作和未成年人保护工作取得进展。侨务、对台、民族宗教、新闻出版、机关事务管理等工作取得新的成绩,老龄、档案、气象、地方志、住房公积金管理等事业有了新的发展。

　　(七)坚持依法行政,服务效能进一步提升。精心组织,严格程序,扎实开展保持共产党员先进性教育活动,构建了“党员长期受教育,群众长期得实惠”的长效机制;广泛听取专家和群众的意见,组织编制了国民经济和社会发展“十一五”规划纲要(草案)。认真贯彻落实《行政许可法》,扩建市行政服务中心,并全面实行5日审批办结制,办事效率有所提高。加强廉政建设,强化审计监督和纠风治乱,深入开展政风评议,促进了机关作风的转变,其中“行风热线”栏目在全国行风建设工作会议上作典型经验介绍。认真听取民主党派、工商联和无党派代表人士的意见,加强了与工会、共青团、妇联、科协等群团组织的联系;自觉接受人民代表大会及其常委会和人民政协的监督,办理人大代表议案和建议114件、政协委员提案197件,群众反映强烈的突出问题得到了较好解决。组织开展村民委员会和社区居委会选举工作,夯实了社会管理的基础。认真做好目标责任的制定、检查和年度考核工作,促进了各项目标任务的完成。

　　去年各项目标任务的顺利完成,为“十五”划上了一个圆满的句号。“十五”以来,我们坚持把加快发展作为第一要务,牢牢把握重要战略机遇期,积极应对新形势,成功克服了非典、洪涝灾害等不利因素的影响,提前实现了“十五”计划确定的主要发展目标。这五年,是经济快速增长、综合实力显著增强的五年。2005年生产总值比2000年翻一番,年均增长10.5%;财政总收入是2000年的2.2倍,年均增长16.6%。这五年,是结构调整成效突出、发展基础不断夯实的五年。累计完成固定资产投资269亿元,是“九五”时期的1.7倍,年均增长15%;支柱产业进一步壮大,农业产业化加快推进,新兴产业加速成长,工业化率达52.5%;新增城市道路面积111万平方米,城市交通环境明显改善,整体功能进一步提升,城镇化率达到50%。这五年,是改革开放进程加快、市场经济体制不断完善的五年。国企改革取得重大进展,雷鸣科化、恒源煤电、飞亚纺织成功上市,4家省属大企业分离办社会职能稳妥推进;非公有制经济不断发展壮大;财政体制改革、农村税费改革、行政管理体制改革进一步深化。对外开放继续扩大,累计完成外贸进出口总额2.3亿美元,是“九五”期间的1.4倍,年均增长10.8%;累计实际利用外资1.2亿美元,是“九五”期间的2.8倍,年均增长21.5%。这五年,还是各项社会事业全面进步、人民群众得到实惠较多的五年。大力实施科技创新,取得科技成果254项;教育事业加快发展,初中阶段适龄人口入学率达到98%,小学学龄儿童入学率接近100%,高等教育、职业教育规模不断扩大;卫生事业长足进步,城乡医疗条件继续改善;文化建设不断加强,相继推出了《大姐》、《大哥》等一批精品力作;群众体育蓬勃发展,竞技体育迈上新台阶;2005年城镇居民人均可支配收入是2000年的1.6倍,年均增长10.1%;农民人均纯收入是2000年的1.3倍,年均增长5%。

　　“十五”的成绩来之不易,积累的经验更是弥足珍贵。我们感到,加快发展、争先进位,必须坚持以科学发展观统揽全局,聚精会神搞建设,一心一意谋发展;必须以工业化为核心,以经济结构调整为主线,大力培育新的经济增长点;必须持续加大投入力度,夯实发展的基础,增添发展的后劲;必须坚持深化改革、扩大开放,以创新激发活力、促进发展;必须坚持以人为本,加快构建和谐社会,实现好、维护好、发展好群众的根本利益;必须切实加强行政效能建设,加速政府职能转变,促进经济社会协调发展。

　　各位代表,“十五”的发展,既使我市站在了一个新的历史起点上,又为实现争先进位、率先崛起奠定了坚实的基础。这是省委、省政府和市委正确领导的结果;是人大、政协监督支持的结果;是全市人民团结奋斗、开拓进取的结果。借此机会,我谨代表市人民政府,向全市各条战线上的广大工人、农民、知识分子、干部、驻淮解放军指战员、武警官兵和政法干警,向市人大代表、政协委员、各民主党派、工商联、人民团体和其他各界人士,致以崇高的敬意!向在我市创业的境内外投资者、建设者,向所有参与、支持、关心淮北建设和发展的同志们、朋友们,表示衷心的感谢!

　　展望“十一五”,随着经济全球化进程不断加快,市场竞争日益激烈,我们面临着巨大的外部竞争压力。从我市经济社会发展的现状来看,也存在着一些薄弱环节:一是经济增长高度依赖煤电产业,地方企业亏损面和亏损额趋于上升。二是对外开放水平不够高,主要是招商引资的总量不够大、质量不够优,开发区建设和发展滞后。三是国企改革任务艰巨,安置职工和解决金融债务的资金净缺口很大。四是全民创业的环境不够优,私营企业数量少、规模小,占全市经济总量的比重较低。五是城乡经济发展不平衡,县区工业经济发展不够快,农村基础设施建设滞后,农民增收的基础不够稳固。同时,经济社会发展中还存在着一些不和谐因素,行政效能有待进一步提高,法制型、服务型政府建设需进一步加强,等等。凡此种种,都要求我们引起高度关注,并正视这些矛盾和问题,在今后的工作中切实加以解决。

　　二、“十一五”时期的奋斗目标和主要任务

　　“十一五”时期是我市争先进位、率先崛起的关键时期。纵观全局,加快全市经济发展,我们面临着难得的机遇。比如,国家实施中部崛起战略和支持资源型城市转型,沿海地区产业、技术、资本加速内移,省委、省政府高度重视“两淮一蚌”煤化工业走廊建设等,有利于我们发挥综合优势,进一步增强发展动力,拓展发展空间。经过多年的探索和实践,我市的发展思路更加清晰、基础更加扎实、氛围更加浓厚,到了应该而且能够率先崛起的时期。我们必须以科学发展观为统领,唱响“争先进位、率先崛起”主旋律,全面推进经济社会跨越式发展。

　　“十一五”时期,全市经济社会发展的指导思想是:坚持以邓小平理论和“三个代表”重要思想为指导,全面贯彻党的十六届五中全会精神,以科学发展观统领经济社会发展全局,以发展为主题,以工业化为核心,以大开放为主战略,以改革创新为动力,以转变经济增长方式为主线,以推进城乡一体化为着力点,以优化环境为保障,加大载体建设力度,加快市场主体培育,加速经济转型,扩张经济总量,提高城市化水平,促进人的全面发展,推动社会全面进步。

　　“十一五”时期,全市经济社会发展的主要目标是:在优化结构和提高效益的基础上,实现社会全面进步和生态环境良好,实现经济总量和人均指标“双翻番”,主要人均经济指标进入全省前6名,加入全省第一方阵。到2010年,全市地区生产总值达到450亿元,年均增长16%左右,人均生产总值达到2万元;财政收入达到56亿元,年均增长17%,人均财政收入达到2500元。

　　“十一五”时期,经济社会发展的主要战略是:坚持推进工业化,实施工业强市战略;坚持招商引资,实施开放带动战略;坚持发展非公有制经济,实施全民创业战略;坚持加快城市化,实施城乡一体化战略;坚持深化改革,实施创新推动战略;坚持以人为本,实施可持续发展战略。 

　　“十一五”时期经济社会发展的主要任务是:

　　一是推进工业结构优化升级,建设现代化工业城市。重点实施“千亿投资计划”,发展煤化工主导产业,做强做大煤电等优势产业;依靠科技进步,改造提升纺织、建材、农副产品加工等传统支柱产业;着眼新的增长点形成,培育发展新兴产业和高新技术产业。

　　二是繁荣农村经济,建设社会主义新农村。按照“生产发展、生活宽裕、乡风文明、村容整洁、管理民主”的要求,坚持“多予、少取、放活”和“工业反哺农业、城市支持农村”的方针,进一步优化农业经济结构,推进农业产业化经营;强化农产品质量管理,完善农业社会化服务体系;加大对农业和农村投入,改善农村基础设施,提高教育、卫生、社保等公共服务水平;建设生态农村,促进可持续发展;扩大劳务输出规模,加快农民向城市和城镇转移。

　　三是扩大消费需求,加快发展现代服务业。积极发展连锁、电子商务等新型业态,提升改造传统商贸业;加快商贸布局调整优化,基本建成布局合理、业态多样、功能齐备、服务优质、消费便利的现代商贸体系。同时,大力推进旅游业、金融保险业、房地产业、社区服务业的发展。

　　四是加快城市化进程,推进城乡一体化。以建设“双百双宜”城市为目标,突破行政区划限制,积极推进市县一体化;加快新城开发和老城改造步伐,强化基础设施建设,提升城市整体功能,集聚产业和人口,发展壮大城区规模;进一步加快小城镇建设;积极争创全国文明城市、生态园林城市、绿化模范城市和环保模范城市。

　　五是积极实施东向战略,大力发展开放型经济。在体制、市场、产业、资本、技术、人才等方面与长三角地区主动对接;强力推进开发区和工业园区建设,完善开发区功能;改进招商方式,优化投资环境,实现招商引资总量、质量的新突破;积极实施“科技兴贸”,优化出口产品结构和贸易业务结构,提高外贸市场竞争力。

　　六是进一步深化改革,全面创新体制机制。积极推进国有企业改革,市属骨干企业全部建立现代企业制度;清理束缚非公有制经济发展的惯例和规章制度,加快非公有制经济发展;深化公共财政体制改革,调整财政支出结构,重点向教育、社保、城乡基础设施建设、农村倾斜;继续推进和完善农村税费改革,粮食流通体制改革,农村信用社改革,投融资体制改革,行政管理体制改革,人事体制和用人机制改革;加快科技创新步伐,促进经济增长方式转变。

　　七是坚持以人为本,构建和谐淮北。继续实施积极的就业政策,大力提升就业水平,切实解决就业困难群体的就业问题。进一步完善社会保障体系,扩大社会保险覆盖范围,健全农村社会养老保险,抓好失地农民的社会保障工作。优先发展教育事业,大力发展文化、体育、卫生和广播电视等社会事业。落实计划生育基本国策,逐步建立利益导向机制。全面落实公民道德建设实施纲要,加强职业道德教育,深入开展“诚信淮北”建设,推动我市文明城市、文明村镇、文明行业“三大创建”共同发展。扎实开展社会治安综合治理,依法严厉打击各类犯罪活动,加快建设“平安淮北”;加强信访工作,认真处理和解决群众反映的问题;加强安全生产工作,强化煤矿等安全整治工作。加强食品药品安全等工作,规范市场秩序。建立健全各种突发性公共安全应急机制和预案,提高政府应对公共突发事件和公共危机的能力。

　　八是发展循环经济,建设环境友好型社会。进一步保护和合理利用土地、矿产资源。加快水源工程建设,进一步推进“淮水北调”工程,解决水资源短缺问题;提高工业用水重复利用率,推广中水的使用和回收;合理开采地下水,减少污水和污染物的排放;加强蓄水和洪水资源利用,推广农业节水灌溉技术。大力发展循环经济,建设节约型社会。加强生态建设和环境保护,建设环境友好型社会。

　　三、扎实做好二○○六年的各项工作

　　2006年是全面实施“十一五”规划的开局之年,也是淮北争先进位、率先崛起的关键之年,做好今年的各项工作,意义十分重大。政府工作的总体要求是:全面贯彻落实党的十六届五中全会和市委五届十一次全会精神,坚持以科学发展观为指导,以发展为主题,以工业化为核心,以大开放为主战略,以改革创新为动力,加快开发区和工业园区建设步伐,实施全民创业行动计划,大力推进城乡一体化,建设现代化工业城市,积极构建和谐淮北,为顺利实施“十一五”规划开好局、起好步。

　　经济社会发展的主要预期目标是:地区生产总值增长12%以上,财政总收入增长12%以上,全社会固定资产投资增长25%,社会消费品零售总额增长10%,城镇居民人均可支配收入增长8%,农民人均纯收入增长8%,人口自然增长率控制在7.7‰以内。

　　围绕上述目标和要求,我们将重点抓好八个方面的工作:

　　(一)突出工业强市,加快建设现代化工业城市。工业是淮北立市的基础和发展的重点,必须认真把握进入重化工业新阶段的机遇,奋力实施工业强市战略,努力实现即期增长与长远发展的统一,速度、质量和效益的统一。

　　以项目建设为重点,夯实发展基础。着力抓好煤化—盐化一体化和南坪煤化工等重大工业项目建设,确保临涣焦化项目部分投产、煤泥矸石电厂开工;开工建设30万吨煤焦油项目、8万吨粗苯项目。加快涡北、孙疃、卧龙湖矿井等重点工程进度,开工建设杨柳、袁店、青东、刘店、邹庄矿井,扩建许疃、任楼矿井;确保恒源煤电矸石电厂、祁东煤电矸石电厂竣工,开工建设500千伏输变电工程等项目,做好平山电厂、国安电厂二期等项目建设的协调工作,增强发展后劲。改造提升纺织、建材、农副产品等传统产业,积极培育生物制药、电子信息、精细化工等新兴产业,进一步增强原有优势产业的规模竞争力。支持县区实施工业强县、工业兴区战略,在重大项目布局上、在发展特色支柱产业上、在培育龙头加工企业上给予政策倾斜,引导县区合理布局、错位发展,加快县区工业化进程。

　　抓好现有工业运行,提高经济效益。我市经济经过连续几年的高位运行,加之新上项目投入大、工期长,短期内很难显现投资效应,继续保持高速增长的压力很大。要紧紧抓住当前宏观经济继续向好,能源价格维持高位、消费需求势头旺盛的市场机遇,积极推动煤电等骨干企业扩大生产销售,继续发挥对经济增长的拉动作用。利用市场和资源约束增强的“倒逼”机制,推动企业联合重组,促进亏损企业扭亏增盈,提高经济运行效益。加强经济运行要素的综合协调,努力化解企业特别是中小企业面临的突出问题,着力改善经济运行环境。

　　(二)统筹城乡发展,加快建设社会主义新农村。“三农”问题始终是争先进位、率先崛起的重点和难点,必须以提高农业综合生产能力、增加农民收入为核心,扎实做好各项工作。

　　继续实施“212强龙工程”,大力推进农业产业化。实施区域化布局、标准化生产、规模化经营,加强100万亩优质麦、30万亩优质棉、200万头生猪等10大优势农业基地建设。重点培育养猪、养禽、食用菌等10大农村专业合作经济组织,提高农民组织化程度。以粮食、畜产品深加工为重点,在资金、用地、税收等方面,重点扶持天宏、鲁王、曦强、正大源、华奥等农副产品加工业龙头企业,促进农产品加工转化增值,加速推进农业产业化。加快农产品质量安全检验检测、农业技术推广、信息服务等五大体系建设,提升服务现代农业发展的能力。

　　落实好各项惠农政策,千方百计增加农民收入。继续巩固农村税费改革成果,全面推进以乡镇机构、农村义务教育和县乡财政管理体制等为主要内容的农村综合改革,创新农村工作机制。稳定农资价格,制止涉农乱收费。改革征地制度,健全对被征地农民的合理补偿机制。积极开发农村劳动力资源,进一步改善农民就业环境,加大对被征地农民的培训力度,全年外出务工农民培训率争取提高5个百分点,输出总量突破30万人。

　　加大农村公共事业投入,扩大公共服务覆盖面。加强农村师资队伍建设,选派城市优秀教师到农村支教,巩固和提高农村九年义务教育水平;落实好国家关于对农村义务教育免收杂费等政策;继续做好农村中小学危房改造工作,改善农村办学条件。发展农村广电事业,建设广播电视“村村通”工程。推进城乡特困群众大病医疗救助和濉溪县新型农村合作医疗试点工作,努力解决农民因病致贫、因病返贫问题。加快推进农村使用沼气和改厕改灶改圈进程,整治农村环境卫生,改善村容村貌。全面启动村村通油路、村村通客车工程,完成270公里乡村道路建设。加强农田水利基础设施建设,发展节水农业,解决2万农村人口饮用水困难。健全村务公开、民主管理制度,有序推进村民自治。

　　(三)扩大消费需求,努力发展现代服务业。消费拉动生产,必须紧紧抓住消费需求趋旺的有利时机,改善消费环境,开拓消费市场。

　　加快发展商贸业。大力发展现代物流业,重点培育与生产企业密切结合的仓储物流业、与电子商务密切结合的批发零售物流业,努力引进外地物流公司,培育骨干物流企业。通过连锁经营、区域性联盟或并购重组等多种方式,推进商贸流通、交通运输等业态创新,提高商贸流通业的现代化水平。

　　大力培育消费需求。积极挖掘文化、体育、教育等市场潜力,引导房地产业健康发展,加快发展城市交通,提升商贸、餐饮业等服务水平,扩大城镇消费。修编旅游发展总体规划,发掘历史文化等旅游资源,加快对南湖湿地公园和相山、龙脊山的开发,建设南湖旅游度假区和双堆烈士陵园、小朱庄等红色旅游景区,扩大旅游消费。大力发展会计、法律、咨询等社会中介服务,努力搞好社区服务,积极培育壮大新兴服务业。

　　切实改善城乡消费环境。加快建设东湖商贸城、亚太购物广场、中瑞农产品批发市场等大市场,盘活、搬迁市工业品批发市场等商品批发市场,进一步集聚人流、物流、信息流。充分运用国家对农村流通和消费市场建设的倾斜政策,抓住濉溪县为省试点、杜集区为市试点的机遇,实施“千乡万村”工程,改善农村消费环境,提高农村消费水平。扎实开展食品放心工程、食品安全信用体系建设等工作,深入开展打击制假售假等市场专项整治活动,进一步整顿和规范市场秩序。

　　(四)推进东向发展,提高对外开放水平。东向发展,核心是优化经济发展空间取向和布局,在更大范围、更高层次上利用两个市场、两种资源,加快外向型经济发展。

　　举全市之力抓好市经济开发区建设。以建设新型工业经济园区和培育新的经济增长极为目标,抓紧做好淮北经济开发区龙湖项目区33平方公里的总体规划和8平方公里起步区控制性规划,今年构建8平方公里起步区基本框架,明年高起点、高标准全面完成“七通一平”建设。认真落实市委、市政府《关于加快开发区建设与发展的实施意见》,对开发区全面简政放权,切实优化建设和发展环境。坚持市、县区统一规划,资源共享,突出特色,进一步加快县区工业园建设。

　　全方位、大力度、宽领域地开展招商引资工作。以工业项目为主,把承接转移与推动产业结构升级结合起来,努力提升招商引资的质量和水平。及时储备更新招商引资项目库,做好项目的推介工作。大力实施资源招商、产业招商、存量招商、中介招商、定向招商和网上招商,重点实行专业招商、定点招商,抽出精兵强将组成专业招商联络组赴沿海各大中城市驻点招商。加大市开发区招商引资力度,对进区项目实行定投资规模、定投资强度、定技术环保要求、定开工竣工时间、定产出效益,重点招大企业、大项目;各县区招商引资项目进入市经济开发区,五年内税收五五分成。推动大企业招商,支持淮矿集团、皖北煤电等大型企业吸引战略投资伙伴,延伸产业链条,形成产业集群。强化招商引资目标管理,严格兑现奖惩,力争全年引进市外资金60亿元人民币,实际利用外资6000万美元。

　　进一步扩大进出口,深化对外交流与合作。积极培育外贸主体,优化外贸产品结构,落实出口扶持政策,转变外贸增长方式,力争进出口总额较快增长。继续推动与韩国抱川市等城市的友好交流,加强与海外华人华侨的交往与联谊,强化与“长三角”和淮海经济区各城市的联系与合作。

　　(五)加快改革创新,增创体制机制新优势。通过创新,为争先进位、率先崛起注入强劲动力。

　　全面完成国企改革任务。集中时间、集中精力、集中资金,强力推进国企改革。纺织一厂、印染集团、热电厂、金贝制衣、燃气公司等12户重点企业要以国有产权退出和职工身份置换为重点,积极筹措改革成本,整合盘活企业资产,妥善处理遗留问题,确保年内全部完成。同时,积极推进事业单位企业化改革和市政公用事业市场化改革,建立健全国有资产监管体系。

　　推进全民创业。认真贯彻落实市委、市政府《关于开展全民创业行动的决定》,进一步加强创业辅导,放宽投资领域,降低市场进入门槛,全面落实优惠扶持政策,通过扶持下岗职工自主创业、鼓励农民致富创业和引导私营个体业主二次创业等,形成百姓创家业、能人创企业、干部创事业的良好局面。建立中小企业信用担保机构,健全信用评价体系,促进银企对接;支持非公有制经济参与国有集体企业改革,投资公共服务领域;引导民营企业加大技改投入,提高产品科技含量;鼓励和推动民营企业通过合资合作等方式,在开放中做大做强;保障非公有制经济的合法权益,提高非公有制经济的比重。

　　加快投融资体系建设。突破“一靠财政、二靠银行”的传统融资模式,大力拓宽融资渠道。整合财政资源,调整资金投向,集中财力用于国企改革和开发区建设;整合土地资源,统一运营市区存量土地,控制土地供应一级市场,以地生财,以地聚财,实现土地资源收益的最大化;整合企业资源,逐步将市属国有企业的有效资产全部纳入市国有资产运营公司管理,实行统一管理、统一重组、统一变现,提高投融资能力;整合党政机关、事业单位经营性资产和文化、体育、教育、卫生、市政公用等部门和单位的特许经营项目,实行招标、拍卖等市场化操作,盘活有形无形资产,开发资源潜在价值,筹集城市建设资金;整合政府信用资源,集中财政资金、社保基金等公共资金,争取银行扩大授信规模。 

　　(六)建设“双百双宜”城市,提升功能品位。适应城市化加速推进的新趋势,推动工业化与城市化相互促进,人口与产业集聚。

　　坚持以规划引领城市发展。积极做好《淮北市城市总体规划》的报批,完成《淮北市近期建设规划(2006—2010年)》的编制工作,扩大控制性详划覆盖率,努力把我市建成“双百”城市和生态山水园林城市。协调并衔接好中心城区与县城、中心镇的建设规划，形成一体化形态格局。抓好村庄规划编制工作，以塌陷区综合治理为契机，加快农村新型社区建设。坚持规划法制化，切实维护规划的严肃性。

　　加快主城区建设。围绕建设“双百双宜”城市和创建全国文明城市、国家环保模范城市、全国生态园林城市、全国绿化模范城市，利用国家开行贷款支持，进一步完善城市路网，改建萧淮路北段，推进南湖路、惠苑东路等在建工程建设，改造惠黎路、长山南路，开工建设张寨路、梅苑路、董庄路、栗苑路、方安路等项目；改造城区供水管网、煤气管网，完成市污水管网一、二期工程建设，加快建设中水回用工程、集中供热工程，开工建设天然气利用工程；加快推进翠峰路立交游园、桓谭公园等项目建设；综合整治化家湖库区，加强塌陷区综合治理，完成长山路沟、跃进河、老濉河、环保沟等城市防洪除涝治理工程；加快市污水处理厂二期、城市垃圾发电厂、医疗废物处置等项目建设进度；增加公交新线路，筹建公交基地停车场和中转调度站，搬迁改建长途汽车客运总站；加快旧城改造，扩展东部城市新区。

　　加强小城镇建设。重点搞好烈山、高岳、百善、临涣、南坪、刘桥等中心镇和建制镇建设；完善小城镇道路、供水、排水、绿化等基础设施建设，净化、美化、亮化城镇环境；增强产业支撑，吸纳集聚人口，提升辐射带动功能，引导矿区职工住宅和采煤村庄搬迁向小城镇集聚。

　　（七）高度关注民生，维护社会稳定和谐。按照“民主法治、公平正义、诚信友爱、充满活力、安定有序、人与自然和谐相处”的要求，解决人民群众最现实、最直接的利益问题。

　　努力扩大就业和社会保障。强化就业目标责任制，全面落实各项优惠政策，重点发展组织起来就业，全年新增城镇就业岗位3万个，多种渠道安置失业人员7000人，城镇登记失业率控制在4%以内。继续巩固“两个确保”，以非公有制经济、灵活就业人员和进城务工人员为重点，做好社会保险扩面征缴工作，启动做实养老保险个人账户，全年养老保险净增1万人，医疗保险净增1万人。完善城市最低生活保障制度，继续做好住房公积金的扩面和廉租房制度推行等工作。加快建立农村低保制度，不断扩大农村低保覆盖面，逐步提高补差标准；高度重视被征地农民的利益，按时全额支付征地补偿安置费用，加强被征地农民就业培训工作，帮助做好再就业工作，并对转为城市户口的，纳入城市居民最低生活保障。

　　提高人民群众文化生活和健康水平。深化文化体制改革、繁荣文化事业、发展文化产业，加强文化、出版物市场管理，加强公共文化设施和基层文化建设，开工建设新图书馆和广电中心大楼，改造全市有线电视网络。继续开展全民健身运动，增强人民体质，力争竞技体育在第十一次全省运动会上争先进位；加快市体育场建设，筹建市游泳馆。积极构建城乡公共卫生和医疗服务体系，高度重视艾滋病、结核病、禽流感等重点传染病的防治，竣工使用市卫生大厦，加快市人民医院康复大楼建设。加强市场监管，维护市场公平公正有序。 

　　保持社会安全稳定。严格落实安全生产责任制和责任追究制，试点推行安全生产抵押金制度；强化安全生产监督管理，努力遏制重特大事故的发生。进一步落实信访工作领导责任制和责任追究制，继续开展领导干部开门接访和带案下访，切实把问题解决在基层、解决在萌芽状态。加强“平安淮北”建设，落实社会治安综合治理措施，严厉打击各类犯罪活动。全面启动“五五”普法。加强减灾防灾工作，完善政府应急体系，强化应急演练，努力提高应对突发事件的能力。

　　（八）推动社会全面进步，促进可持续发展

　　实施科教兴市、人才强市战略。围绕加快科技创新，加大科技投入，加快建设科技企业孵化器，搞好民营科技园建设，积极筹建淮北煤化工研发基地，依靠先进适用技术改造提升传统产业，提高企业自主创新能力。重视提高义务教育质量，全面推进素质教育；建立健全农村义务教育投入保障机制，鼓励民资办学；合理调整中小学布局，努力改善薄弱学校办学条件；开工建设投资５０００万元、九年一贯制的梅苑中学；积极支持淮北煤师院和淮北职业技术学院建设，扩大院校规模，提升综合办学水平；大力发展职业教育，促进基础教育、职业教育和成人教育统筹协调发展。把人才强市战略放在重要位置，积极引进人才，用好现有人才，培养后备人才，健全有利于人才资源发挥作用的体制机制，努力把我市建成人才辈出、人尽其才、才尽其用的高地。

　　建设资源节约型和环境友好型社会。推行清洁生产，大力发展循环经济，提高资源综合利用效率。综合运用价格等政策，开展节能、节地、节水、节约原材料活动，降低消耗。坚持节约集约利用土地，盘活闲置存量土地，合理利用采煤塌陷地，保障项目用地特别是重大工业项目建设用地，保持耕地总量占补平衡。扎实推进全国节水型社会试点市建设，加强水资源保护和利用，建设一批蓄水、储水工程，推进“淮水北调”工程和临涣工业园引水工程建设。深化全民义务植树运动，加快封山育林步伐，提高森林覆盖率，推动绿色城市、绿色厂矿、绿色村庄、绿色家园建设，打造“生态淮北”。 

　　加快发展各项社会事业。加强人口与计划生育工作，创新工作机制，强化保障措施，改进考核办法，突破农村和城市计生工作的重点、难点，努力实现争先进位。进一步强化统计预警监测职能，积极做好第二次农业普查工作。抓好外事、侨务和对台事务。做好档案、气象、防震、文物、地方志、新闻出版等工作。重视妇女儿童、老龄和残疾人事业，加强社会福利和老年服务设施建设。做好民族宗教工作。深化人防知识教育。加强国防动员和后备力量建设，支持驻淮部队和武警部队建设，做好淮北军分区搬迁工作，深入开展“双拥”活动，巩固军政军民团结。

　　四、进一步加强政府自身建设

　　做好今年经济社会发展的各项工作，实现“十一五”发展开好局、起好步，对政府提出了新的更高的要求，我们必须以全新的思路、全新的举措，切实加强政府自身建设。

　　振奋精神，建设务实政府。深入开展以“转变观念、提高效能”为主题的解放思想主题实践活动，切实把各级政府工作人员的思想统一到党的十六届五中全会和市委五届十一次全会精神上来，把精力集中到加快发展上来。进一步强化责任意识和大局意识，坚持敢于碰硬、勇于负责，主动排难而进；进一步坚持求真务实、雷厉风行，快速推进各项工作；进一步坚持奋力赶超、争创一流，在争先进位、率先崛起中建功立业。 

　　转变职能，建设高效政府。积极推进政企分开、政资分开、政事分开，政府与市场中介组织分开，减少和规范行政审批，简政放权，把政府职能切实转到经济调节、市场监管、社会管理和公共服务上来。完善行政管理决策机制，健全重大事项决策的协商协调机制，健全对专业性、技术性较强重大事项决策的专家论证、技术咨询、决策评估制度，健全对与群众利益密切相关重大事项决策的公示、听证制度，推进行政管理决策科学化、民主化。进一步完善行政服务中心的功能，落实五日办结制，提高行政效率。继续创新政府工作流程，加强电子政务建设，大力精简会议和文件，减少事务性应酬，降低行政成本。加强行政效能监察，切实解决少数地方和部门存在的本位主义、推诿扯皮，甚至吃拿卡要等问题，强化执行能力，确保政令畅通。

　　依法行政，建设法制政府。落实《全面推进依法行政实施纲要》，强化行政执法责任制和执法过错追究制，认真解决有法不依、执法不公和多头执法、执法扰民等问题，保证各级行政管理机关及其工作人员严格按照法定权限和程序行使职权、履行职责，做到有权必有责、用权受监督、侵权要赔偿、违法要追究。认真落实市人大及其常委会的各项决议和决定，坚持重大事项报告制度，自觉接受法律监督和工作监督。认真听取政协及各民主党派、工商联、无党派代表人士的意见，自觉接受民主监督。认真办理人大议案、建议和政协提案。主动加强与工会、共青团、妇联等社团组织的联系，接受群众监督和舆论监督，不断改进政府工作。 

　　从严治政，建设廉洁政府。认真落实党风廉政建设责任制，建立和完善惩防并举的反腐倡廉机制，建立体现科学发展观和正确政绩观要求的干部实绩考核评价制度，健全干部任期经济责任审计等制度，切实加强廉政建设。继续采取行之有效措施，坚决纠正部门和行业不正之风。继续保持艰苦奋斗的优良作风，大力开展节约型机关创建活动，反对奢侈浪费，把有限的资金用到促进发展上，用到改善人民群众生活上。

　　各位代表，实现淮北争先进位、率先崛起，是时代赋予我们的光荣使命。让我们在市委的坚强领导下，在市人大、市政协的监督支持下，以邓小平理论、“三个代表”重要思想和科学发展观为指导，全面贯彻党的十六届五中全会、中央经济工作会议和市委五届十一次全会精神，团结一致、抢抓机遇，脚踏实地、开拓创新，为“十一五”开好局、起好步而努力奋斗。
